# Popliteal Artery Aneurysm Thrombosis Diagnosed with Point-of-Care Ultrasound

**DOI:** 10.5811/cpcem.52832

**Published:** 2026-08-05

**Authors:** Andrew Gonedes, Brian Kohen, Alfa Diallo, Mark McKenna, Robert Farrow, Eric Boccio

**Affiliations:** * Memorial Healthcare System, Department of Emegency Medicine, Hollywood, Florida; † Mount Sinai Medical Center of Florida, Department of Emergency Medicine, Miami Beach, Florida

**Keywords:** popliteal artery aneurysm thrombosis, point-of-care ultrasound, arterial thrombosis, acute limb ischemia, vascular emergency

## Abstract

**Case Presentation:**

A 62-year-old male presented to the emergency department with a three-day history of right knee, calf, and foot pain following a bout of prolonged crouching while gardening. The physical examination revealed a cold and pale foot with absent pulses. A point-of-care ultrasound (POCUS) performed at bedside revealed a thrombosed popliteal artery aneurysm. A heparin infusion was initiated, and vascular surgery was consulted. Subsequent imaging confirmed the diagnosis of a thrombosed popliteal artery aneurysm with thrombus extension into the superficial femoral artery. The patient underwent a femoropopliteal bypass, resulting in successful revascularization of the limb.

**Discussion:**

While POCUS is a well-established imaging modality for the diagnosis of deep vein thrombosis, its utility in diagnosing acute peripheral arterial pathologies is less well-known. This case demonstrates how prompt bedside POCUS evaluation can circumvent potential delays associated with traditional imaging such as computed tomography angiography, allowing for expedited surgical consultation and timely therapeutic and procedural intervention. This case highlights the potential role of POCUS in the rapid diagnosis of acute limb ischemia from arterial thrombosis in the acute care setting.

## CASE PRESENTATION

A 62-year-old man presented to the emergency department (ED) with a three-day history of escalating pain in his right knee, calf, and foot. The pain initially began behind his right knee after a prolonged period of crouching while gardening. Over the subsequent 72 hours, the discomfort progressively worsened, radiating distally down his calf and into his foot, which prompted his visit to the ED. On physical examination, the right lower extremity was cold, exhibited significant pallor, and had decreased sensation. Dorsalis pedis and posterior tibial pulses could not be palpated, and a pulse Doppler also failed to detect any audible flow.

Given the limb’s appearance on physical examination, an acute arterial pathology was highly suspected. An emergency physician immediately performed point-of-care ultrasound (POCUS) beginning in the popliteal fossa, which revealed a fusiform popliteal artery aneurysm with no Doppler flow (Video 1). A thrombus was found to extend to the right superficial artery on subsequent images (Videos 2 and 3). An intravenous infusion of unfractionated heparin was initiated, and vascular surgery was consulted emergently. Computed tomography angiography of the right lower extremity was confirmatory. The patient underwent emergent right femoropopliteal bypass surgery, which resulted in successful revascularization and preservation of the right lower extremity.

## DISCUSSION

Popliteal artery aneurysm thrombosis is a vascular emergency that can result in acute limb ischemia if not promptly recognized. Point-of-care ultrasound provides a rapid, bedside diagnostic tool that can guide early management. Using a high-frequency linear probe, the popliteal fossa is scanned in both transverse and longitudinal planes. A normal popliteal artery should appear as a round, anechoic, pulsatile structure, with color Doppler demonstrating laminar flow. In the setting of an aneurysm, the vessel diameter is dilated beyond 2 cm, often with mural thrombus visible as echogenic material within the lumen. Complete thrombosis is suggested by absence of color Doppler flow within the aneurysmal segment, while partial thrombosis may reveal heterogeneous echogenic areas interspersed with residual flow channels. Spectral Doppler can further demonstrate dampened, monophasic, or absent waveforms distal to the occlusion, supporting the diagnosis of impaired arterial perfusion.

The accuracy of POCUS is well documented for certain vascular pathologies such as deep vein thrombosis, and POCUS is particularly valuable in differentiating thrombotic occlusion from embolic phenomena or nonaneurysmal peripheral arterial disease.^1^ The lack of established guidelines for POCUS use in arterial thrombosis highlights a current gap in emergency medicine practice. Nonetheless, prior case reports have demonstrated the utility of POCUS in detecting acute peripheral arterial pathologies.^2^–^4^ Bedside detection allows for expedited vascular surgery consultation, early initiation of anticoagulation, and timely revascularization planning. While computed tomography angiography remains the gold standard, POCUS offers a rapid, noninvasive, and repeatable method for identification of popliteal artery aneurysm thrombosis in the acute care setting.

*CPC-EM Capsule*

What do we already know about this clinical entity?

*Popliteal artery aneurysm thrombosis is a vascular emergency that can cause acute limb ischemia and requires rapid diagnosis to prevent limb loss.*

What is the major impact of the image(s)?

*The images demonstrate the utility of point-of-care ultrasound (POCUS) in identifying aneurysmal dilation and thrombus.*

How might this improve emergency medicine practice?

*Prompt POCUS evaluation allows for expedited surgical consultation and earlier anticoagulation by bypassing delays from traditional imaging.*

## Supplementary Information

Video 1 Thrombosed popliteal artery aneurysm with absence of pulsatile and color Doppler flow.

Video 2 Extension of thrombus to the superficial femoral artery (A) alongside the femoral vein (V) seen in long-axis view.

Video 3 Extension of thrombus to the superficial femoral artery (A) alongside the femoral vein (V) seen in short-axis view.
